# Discrediting experiences: outcomes of eligibility assessments for claimants with psychiatric compared with non-psychiatric conditions transferring to personal independence payments in England

**DOI:** 10.1192/bjo.2019.3

**Published:** 2019-02-04

**Authors:** Katie Pybus, Kate E. Pickett, Stephanie L. Prady, Charlie Lloyd, Richard Wilkinson

**Affiliations:** Research Fellow, Department of Health Sciences, University of York, UK; Professor of Epidemiology, Department of Health Sciences, University of York, UK; Senior Research Fellow, Department of Health Sciences, University of York, UK; Reader in Social Policy and Criminal Justice, Department of Social Policy and Social Work, University of York, UK; Professor Emeritus, University of Nottingham Medical School; Honorary Professor, University College London; and Honorary Professor, Department of Health Sciences, University of York, UK

**Keywords:** Service users, parity of esteem, personal independence payments, welfare reform, eligibility assessment

## Abstract

**Background:**

Recent qualitative research suggests that changes to the way eligibility for welfare payments is determined in the UK may be detrimental to claimants with mental illnesses. No large-scale analysis has been undertaken to date.

**Aims:**

To examine differences between claimants with psychiatric conditions compared with non-psychiatric conditions in the number of claims disallowed following a personal independence payment (PIP) eligibility assessment for existing disability living allowance (DLA) claimants.

**Method:**

Administrative data on DLA claimants with psychiatric conditions transferring to PIP between 2013 and 2016 was compared with claimants with non-psychiatric conditions to explore differences in the number of claims disallowed following an eligibility assessment.

**Results:**

Claimants with a mental illness were 2.40 (95% CI 2.36–2.44) times more likely to have their existing DLA entitlement removed following a PIP eligibility assessment than claimants with musculoskeletal conditions, neurological conditions and diabetes.

**Conclusions:**

PIP eligibility assessment outcomes show marked differences by health condition, raising questions as to whether the process is equitable.

**Declaration of interest:**

None.

Since the introduction of disability living allowance (DLA) in 1992, the assessment of eligibility for disability-related social security payments in the UK has undergone several iterations.^1^ Most recently there has been a large-scale overhaul of the existing system and a new replacement extra-costs disability benefit, the personal independence payment (PIP), has been introduced.

The initial rollout of PIPs for existing DLA claimants took place from 2013 onwards in England. In the first instance, working age DLA claimants were invited to apply for PIP where a change in circumstances was likely or had arisen: if the Department for Work and Pensions (DWP) was notified of a change in care or mobility needs; the claim had a fixed term that was due to expire; a child in receipt of DLA had turned 16; or the claimant voluntarily chose to transfer to PIP. The rollout was expanded so that by 2015 most existing DLA claimants had been invited to undergo reassessment.^2^

The key changes to eligibility assessments for PIPs compared with DLA for most claimants include a longer qualifying period, mandatory periodic claim reviews and additional requirements to attend a face-to-face medical assessment.^3^ The updated functional assessment also takes account of aids and adaptations when considering how a person is affected by their health condition.^4^ Aiming to streamline the 11 different levels of payment available under DLA and to offer greater clarity for claimants, PIP is awarded based on a person needing help for more than half of the time in two main areas: daily living and mobility.^3^^,^^5^ Both components can be awarded at a standard or enhanced rate depending on how severely the person is assessed as being affected by their health condition.^6^ Once an initial paper application has been completed, claimants are invited to an eligibility assessment conducted by contractors (for example Capita, ATOS) and information is then passed to a DWP ‘decision-maker’ who decides on the final award.

Although designed to simplify the claims process and target financial support more effectively for those people with disabilities most in need, the implementation of PIP has been accompanied by controversy. Concerns have been raised by disability charities that the 20% savings target attached to the reforms is arbitrary rather than being grounded in evidence relating to levels of need among the disabled population.^3^ An independent review carried out following implementation of PIP suggests that the new eligibility assessment process fails to fully understand the challenges faced by individuals with disabilities, resulting in difficulties accessing the appropriate level of financial entitlement.^7^

## Eligibility assessments and mental illness

Although the new eligibility assessments have been problematic for a wide range of claimants and a stated aim of the change from DLA to PIP is to cut spending, people with mental health conditions do appear to have been disproportionately affected by the reforms. Some individuals have seen higher levels of payment; however, overall the reduction in eligibility appears to have been particularly concentrated on those with mental health conditions.^8^^,^^9^ In March 2017, for example, the UK government announced that enhanced-rate mobility payments as part of PIP would no longer be made to claimants on the grounds of ‘psychological distress’, meaning that only physical mobility difficulties would be eligible for the higher rate of financial award. A high court ruling in December 2017 described this restriction as ‘blatantly discriminatory’ against people with mental illness.^10^^,^^11^

Welfare reform more broadly appears to have been especially problematic for people with mental illnesses. Compared with other claimant groups, people with mental health problems are more likely to face benefit sanctions^12^ and in recent qualitative research have described experiences during eligibility assessments of their difficulties being trivialised or viewed as altogether fraudulent.^13^^,^^14^ There may be questions about apparent ‘parity of esteem’ for mental and physical health conditions in the welfare system. In this study, we aimed to explore whether differences exist in PIP outcomes between existing DLA claimants with psychiatric compared with non-psychiatric conditions. We focus on outcomes following the eligibility assessment component of the PIP claims process.

## Method

### Data

Publicly available administrative data from the DWP was downloaded on 22 September 2017. The data extracted for the analysis covers all claimants aged 16–64 who had been reassessed from an existing DLA entitlement for transfer to PIP between April 2013 and October 2016.^2^ Reassessment data and not data for new claimants were selected for this study because this provides an indication of an existing need that has previously enabled the claimant to successfully obtain state financial support. It also offers the opportunity to compare old and new disability payment systems across different health conditions.

The statistical release provides count data for total number of claims alongside the proportion of claimants experiencing each of the following reassessment outcomes: (a) award increased, (b) award decreased, (c) award unchanged, (d) award disallowed pre-referral to the assessment providers (claim turned down based on the paper application – failure to meet basic eligibility criteria or to return part of the form), (e) claim withdrawn and (f) award disallowed post-referral to the assessment providers (claim disallowed following an eligibility assessment). We classified outcome (f) against all other outcomes to test the likelihood of a claimant having their existing DLA entitlement removed following an eligibility assessment compared with having any other reassessment outcome.

Count data was derived from the reported proportion of claimants experiencing each of the six reassessment outcomes by each health condition. The outcome for each group was then categorised as ‘claim disallowed following an eligibility assessment’ versus all other outcomes. As a result of the rounding up of proportions occurring at source, there is a slight variance between the overall number of claimants reassessed and the converted count data for each reassessment outcome. This is between 0% and 1% for all health conditions and reassessment outcomes in the sample. There were no missing data for any of the variables used in this analysis.

Claimants in this data-set are categorised by main health condition; the primary reason for the existing DLA claim. Six psychiatric conditions were selected for use in the analysis to give a broad range of different mental illnesses. The original categories of DLA claims have been retained here to enable replication of findings: psychosis, personality disorder, psychoneurosis, behavioural disorders, alcohol and drug use and hyperkinetic syndrome. Psychoneurosis and hyperkinetic syndrome will, however, be referred to by the terms ‘anxiety and mood disorders’ and attention-deficit hyperactivity disorder (ADHD) respectively. An aggregated variable incorporating all of these categories, titled ‘all psychiatric conditions’, was also derived for use in the analysis.

Three categories of non-psychiatric conditions were used as comparators. A ‘musculoskeletal conditions’ variable including claimants listing arthritis, back pain, diseases of muscles, bones and joints or spondylosis, a ‘neurological conditions’ comparator incorporated multiple sclerosis and epilepsy and a third category of people with diabetes were derived from the data. These conditions were selected to represent a range of commonly reported, visible and non-visible, chronic and potentially relapsing health problems with the aim of providing, as far as possible, some comparability with psychiatric conditions. An ‘all non-psychiatric conditions’ variable was also created incorporating all of these comparators.

### Statistical analysis

The likelihood of having a claim for PIP disallowed following an eligibility reassessment compared with any other outcome was estimated for those with psychiatric conditions (exposed) relative to each of the non-psychiatric comparators (unexposed). We calculated odds ratios and reported 95% CIs from the derived count data using the immediate command ‘cci’ in Stata v15.1.

## Results

Approximately 148 700 claimants listing a psychiatric condition and 178 300 claimants listing a non-psychiatric condition were included in the analysis (Table 1). A total of 32% (*n* = 47 741) of claimants with psychiatric conditions lost their existing financial entitlement following a PIP eligibility assessment between 2013 and 2016 compared with 16.4% (*n* = 29 323) of those with a non-psychiatric condition.

**Table 1:** Descriptive statistics for reassessment outcomes by health condition^a^

Health condition	Claim disallowed following eligibility assessment, *n* (%)	All other reassessment outcomes, *n* (%)	Total claimants, *n*
All psychiatric conditions	47 741 (32.1)	100 959 (67.9)	148 700
Psychosis	22 661 (31.0)	50 439 (69.0)	73 100
Personality disorder	1953 (31.0)	4347 (69.0)	6300
Psychoneurosis (anxiety and mood disorders)	17 391 (33.0)	35 309 (67.0)	52 700
Behavioural disorders	1120 (32.0)	2380 (68.0)	3500
Alcohol and drug use	1456 (28.0)	3744 (72.0)	5200
Hyperkinetic syndrome (ADHD)	3160 (40.0)	4740 (60.0)	7900
All non-psychiatric conditions	29 323 (16.4)	148 977 (83.6)	178 300
Musculoskeletal conditions	20 226 (13.7)	127 674 (86.3)	147 900
Neurological conditions	7392 (29.7)	17 508 (70.3)	24 900
Diabetes mellitus	1705 (31.0)	3795 (69.0)	5500

ADHD, attention-deficit hyperactivity disorder.

a. Numbers are subject to rounding errors (see Method).

Overall, claimants with a psychiatric condition were 2.40 (95% CI 2.36–2.44) times more likely than a claimant with a non-psychiatric condition to have their existing DLA entitlement removed following a PIP eligibility assessment (Table 2). This ranged from 1.97 (95% CI 1.85–2.10) times more likely for claims based on alcohol and drug use, to 3.38 (95% CI 3.23–3.55) times more likely for claimants with ADHD.

**Table 2:** Odds ratios for claims disallowed following an eligibility assessment during reassessment from disability living allowance to personal independence payment for psychiatric conditions compared with non-psychiatric conditions

	OR (95% CI)						
	All psychiatric conditions	Psychosis	Personality disorder	Psychoneurosis	Behavioural disorder	Alcohol and drug use	ADHD
All non-psychiatric conditions	2.40 (2.36–2.44)***	2.28 (2.23–2.32)***	2.28 (2.15–2.41)***	2.50 (2.44–2.55)***	2.39 (2.22–2.57)***	1.97 (1.85–2.10)***	3.38 (3.23–3.55)***
Musculoskeletal conditions^a^	2.98 (2.93–3.04)***	2.83 (2.77–2.89)***	2.83 (2.68–2.99)***	3.10 (3.03–3.18)***	2.97 (2.76–3.19)***	2.45 (2.30–2.61)***	4.20 (4.01–4.41)***
Neurological conditions^b^	1.12 (1.08–1.15)***	1.06 (1.03–1.09)***	1.06 (1.00–1.13)*	1.16 (1.12–1.20)***	1.11 (1.03–1.20)**	0.92 (0.86–0.98)*	1.57 (1.49–1.66)***
Diabetes	1.05 (0.99–1.11)	1 (0.94–1.06)	1 (0.92–1.08)	1.09 (1.03–1.16)*	1.04 (0.95–1.14)	0.86 (0.79–0.94)***	1.48 (1.37–1.59)***

ADHD, attention-deficit hyperactivity disorder.

a. Musculoskeletal conditions includes arthritis, back pain, diseases of muscles, bones and joints, spondylosis.

b. Neurological conditions includes epilepsy and multiple sclerosis.

* *P* < 0.05, **P < 0.01, ***P < 0.001.

Claimants with a common mental disorder such as anxiety or depression were more likely to have their claim disallowed than claimants with any of the non-psychiatric conditions included in the analysis; ranging from 1.09 (95% CI 1.03–1.16) times more likely compared with people with diabetes to 3.10 (3.03–3.18) times more likely compared with people with musculoskeletal conditions.

Claimants with personality disorder or psychosis were more likely to have their claims disallowed compared with all other non-psychiatric groups except people with diabetes, compared with whom they were just as likely to have their claims disallowed.

The likelihood of having a claim disallowed for claimants with a behavioural disorder ranged between 1.11 (95% CI 1.03–1.20) compared with claimants with neurological conditions and 2.97 (95% CI 2.76 to 3.19) for claimants with musculoskeletal conditions. Individuals citing alcohol and drug use as the main reason for their claim were less likely than claimants with neurological conditions to have their claim disallowed (0.92, 95% CI 0.86–0.98) but more likely than those with musculoskeletal conditions (2.45, 95% CI 2.30–2.61). Of all the groups with psychiatric conditions, claimants with ADHD had the highest likelihood of having their claim disallowed compared with any of the non-psychiatric groups.

There was little variation in the likelihood of having a claim disallowed for individuals with psychiatric conditions compared with those with diabetes, aside from alcohol and drug users who were less likely (0.86, 95% CI 0.79–0.94), whereas claimants with ADHD (1.48, 95% CI 1.37–1.59) and anxiety or depression (1.09, 95% CI 1.03–1.16) were more likely to have their claim disallowed following a PIP eligibility assessment.

## Discussion

Although previous campaigning organisations have analysed these data^8^^,^^9^ as far as we are aware, this is the first academic study to analyse data on the outcomes of benefit reassessments in the UK. The findings suggest that in general, the number of claims disallowed following a PIP eligibility assessment is elevated for psychiatric conditions compared with non-psychiatric conditions with variations by type of mental illness. People with diabetes were the exception, where rates of disallowed claims were similar to those of people with psychiatric conditions. However, this is not necessarily an encouraging finding given that the proportion of claims disallowed for psychiatric conditions accounts for at least a third of the reassessment outcomes for this group.

Further to these findings using relative estimates, it is also worth highlighting that the number of claimants affected in absolute terms is far higher for mental illness than for other health conditions. Overall, 47 741 claimants with a psychiatric condition had their existing DLA entitlement removed after undergoing a PIP eligibility assessment between 2013 and 2016. This is a substantial number of people now without financial support to which they previously had access.

It is not clear from this study alone why individuals with mental health conditions appear to be at a disadvantage compared with claimants with some other types of health condition or whether the findings are related to other factors, for example, the visibility of an illness; however, these findings do support existing concerns about the migration from DLA to PIP for people with mental illnesses.^9^ Testimony from PIP claimants gathered recently by the House of Commons Work and Pensions Committee^15^ detailing experiences of eligibility assessments highlighted issues such as a lack of knowledge about the impact of mental illnesses on daily functioning on the part of assessors, coupled with the use of ‘informal observation’, for example, appearance and body language, to make broad inferences about the mental state of the claimant. Assessment providers giving written evidence to the same inquiry in response to a question about whether the professional background of assessors is matched to the health condition experienced by the claimant have stated that ‘Our role is not to diagnose or treat so specialist knowledge of, for example, mental health diagnosis and treatment is not necessary to be able to understand how an individual's life is affected’.^16^ As of November 2017, for example, 16.6% of ATOS PIP assessors had a clinical mental health background.^16^ Our study contributes empirical evidence to the ongoing debate about whether the PIP claims process is fit for purpose in its current form.

This study focuses on existing DLA claimants but does not consider the outcomes of new claimants of PIP who have not previously sought financial support for their mental health condition. The political response to the recent mobility payments ruling is that all those currently in receipt of PIP will have their claim reassessed and backdated payments provided where required.^17^ This decision does not, however, appear to take account of all those who may have had their claim disallowed entirely at the eligibility assessment phase because they did not gain enough points to meet the criteria for payments since their mobility needs were not included. Further research, using data from March 2017 onwards, when rules on mobility payments for psychological distress were implemented, is needed to examine whether these changes had any impact on the number of claims disallowed.

### Implications of findings

Our study indicates the possibility that PIP eligibility assessments may have a negative impact on claimants with psychiatric conditions. These findings echo those of recent qualitative studies undertaken in the UK in which claimants with mental illness have described feeling disadvantaged by their health condition during the eligibility assessment process.^13^^,^^14^ The existing relationship between mental illness and disadvantage is well-established^18^ and seemingly difficult to shift. The loss of further income, up to £141.10 per week for people with the most severe conditions, has potential implications for increasing such inequalities and with it the increased risks of morbidity and mortality attached to living in poverty. Recent research suggests that people with mental illnesses are overrepresented as food bank users compared with the general population,^19^ pointing to heightened financial difficulties. With welfare payment claims for mental health conditions rising over time^20^ and with increasingly larger numbers of people affected, an effective and fair resolution is needed.

This study has considered psychiatric conditions relative to other health conditions to explore differences in reassessment outcomes. The intention is not to suggest that the proportion of claims disallowed for non-psychiatric conditions is at an acceptable level but rather to highlight potential areas of disadvantage that may need addressing to ensure equitable access to financial support, based on need, for all disabled people. Parity of esteem between mental and physical health has received sustained attention in recent years, most notably being enshrined in law for health outcomes in the National Health Service.^2^,^1^ Our findings raise the question of whether the parity of esteem agenda should be extended to cover other public institutions such as the welfare system because if equity is not achieved, people with mental illnesses risk becoming even more marginalised as a result of their health conditions.

### Limitations

From these aggregated data it was not possible to determine an independent baseline level of health-related needs for claimants undergoing reassessment of their existing DLA entitlement or to incorporate individual characteristics such as age or gender into the analysis. It is feasible, for example, where health conditions are chronic and remitting, as may be the case for some psychiatric illnesses, that the existing level of financial support was not required at the point of reassessment. This is also true across some of the comparator conditions in the analysis (for example people with back pain) and raises questions as to why claimants would choose to undergo a reassessment with the associated stress this may entail, if no longer in need of financial support. Nevertheless, the data are for individuals with an identified health-related need, previously assessed independently as being at a level requiring financial support.

The comparators used in this analysis are by necessity broad and do not reflect the variation that could be associated with individual illnesses; however, the study sought to compare different types of non-psychiatric condition and conditions were carefully selected to represent illnesses that could be remitting, relapsing and potentially not visible. The findings here focus on one specific reassessment outcome, claims disallowed following an eligibility assessment. Further analysis could explore other reassessment outcomes by health condition, for example, the number of claims that were awarded following an eligibility assessment but where the financial entitlement has been either increased or decreased, particularly given that this has provided justification for changes to the existing payment system. Consideration should also be given as to why individuals with diabetes experience similar proportions of disallowed claims to people with mental illness. It was also not possible to disaggregate the psychoneurosis category by type of illness; therefore, it is possible that a whole spectrum of claimants is represented here, from milder forms of dysthymia through to severe depressive illness, with potentially differing claim outcomes. If data were provided that facilitated such an analysis, future research could focus on exploring outcomes by severity of illness.

The rollout of PIP reassessments did not occur uniformly, and this may have implications for outcomes over time between 2013 and 2016. Reassessment was undertaken based on the type of claim rather than incorporating, for example, all individuals claiming DLA in a particular postcode, and so the likelihood of the data being influenced by the characteristics of an area is low, but it is possible that learning over time from these early assessments could have influenced later outcomes. Any alterations to the process may have acted either in favour or against claimants with mental illnesses who were assessed further along in the implementation period.
