# Discrediting experiences: outcomes of eligibility assessments for claimants with psychiatric compared with non-psychiatric conditions transferring to personal independence payments in England – ERRATUM

**DOI:** 10.1192/bjo.2019.16

**Published:** 2019-03-05

**Authors:** Katie Pybus, Kate E. Pickett, Stephanie L. Prady, Charlie Lloyd, Richard Wilkinson

**Keywords:** Service users, parity of esteem, personal independence payments, welfare reform, eligibility assessment, erratum

The publishers regret to announce that the above paper was published with the incorrect article number.

The correct citation details are as follows:

Pybus K, Pickett KE, Prady SL, Lloyd C, Wilkinson R. Discrediting experiences: outcomes of eligibility assessments for claimants with psychiatric compared with non-psychiatric conditions transferring to personal independence payments in England. *BJPsych Open* 2019; 5(2): e19. https://doi.org/10.1192/bjo.2019.3.

This has now been updated in the original article online.

The publisher sincerely apologises for this error.
